# Predictive value of uric acid-to-high-density lipoprotein cholesterol ratio for cardiometabolic multimorbidity in middle-aged and older adults: A nationwide prospective cohort study

**DOI:** 10.1097/MD.0000000000049740

**Published:** 2026-07-10

**Authors:** Shijing Jiang, Shuliang Wang, Zhiwei Miao

**Affiliations:** a Department of Traditional Chinese Medicine, Taixing Second People’s Hospital, Taixing, Jiangsu, China; b Department of Gastroenterology, Zhangjiagang TCM Hospital Affiliated to Nanjing University of Chinese Medicine, Zhangjiagang, Jiangsu, China.

**Keywords:** cardiometabolic multimorbidity, CHARLS, high-density lipoprotein cholesterol, uric acid

## Abstract

Cardiometabolic multimorbidity (CMM) is an escalating public health challenge. The uric acid (UA)-to-high-density lipoprotein cholesterol (HDL-C) ratio (UHR) is a composite biomarker reflecting metabolic disturbance, but prospective evidence regarding the association between UHR and CMM remains limited. This prospective cohort study included 7435 adults aged ≥ 45 years from China Health and Retirement Longitudinal Study followed from 2011 to 2018. Cox proportional hazards models and restricted cubic spline analyses were used to examine the associations of UHR and cumulative UHR (CumUHR) with CMM. Receiver operating characteristic curves, net reclassification improvement, and integrated discrimination improvement were used to compare the incremental predictive performance of UHR and CumUHR with that of UA and HDL-C alone. Subgroup and sensitivity analyses were conducted to test the robustness of the findings. Among the 7435 participants, 1748 developed CMM. Kaplan–Meier analysis showed that the cumulative event rate of CMM increased progressively across UHR quartiles (log-rank *+* < .001). In the fully adjusted model, the highest UHR quartile (Q4) was associated with a significantly increased risk of CMM compared with the lowest quartile (Q1) (hazard ratio = 1.48, 95% confidence interval 1.27–1.77). When cumulative exposure was considered, elevated CumUHR remained an independent predictor of CMM (hazard ratio = 1.26, 95% confidence interval 1.15–1.39). Restricted cubic spline analyses further demonstrated significant nonlinear associations of both UHR and CumUHR with CMM risk, with inflection points observed around 8.5 for UHR and 35.7 for CumUHR. Furthermore, UHR and CumUHR showed better predictive performance for CMM than their individual components, with higher areas under the curve and significant improvements in net reclassification improvement and integrated discrimination improvement, whereas UA and HDL-C alone did not significantly improve these predictive indices. Higher UHR and CumUHR levels were independently associated with an increased risk of CMM in middle-aged and older adults. As composite indicators integrating UA and HDL-C, UHR and CumUHR may provide complementary information for cardiometabolic risk assessment and may help identify individuals at elevated risk of CMM when considered alongside established clinical risk factors.

## 1. Introduction

Cardiometabolic multimorbidity (CMM) refers to the coexistence of at least 2 cardiometabolic diseases (CMDs) within the same individual, commonly including heart disease, stroke, diabetes, and hypertension.^[1]^ With population aging and the rising prevalence of metabolic risk factors, CMM has become an increasingly important public health challenge. In a nationwide cohort of 512,723 Chinese adults, 15.8% of participants had multimorbidity overall, and 6.0% specifically exhibited CMM.^[2]^ Beyond its high prevalence, CMM evolves through the progressive accumulation of CMDs over time. In a large prospective cohort of 461,047 adults followed for more than 11 years, participants transitioned sequentially from a healthy state to a first CMD and subsequently to CMM, with high-risk lifestyle factors accelerating these transitions.^[3]^ Early identification of individuals at high risk of developing CMM is therefore essential. In this context, identifying simple and accessible biomarkers that reflect long-term cardiometabolic burden is of considerable clinical interest.

Uric acid (UA) has been consistently associated with increased cardiometabolic risk.

A large prospective cohort study in Chinese adults showed that urate-related disorders were associated with a substantial comorbidity burden, including increased risks of cardiovascular disease (CVD), chronic kidney disease, diabetes, and all-cause mortality.^[4]^ Meanwhile, high-density lipoprotein (HDL) cholesterol (HDL-C) is widely recognized for its protective role in lipid metabolism and vascular health.^[5]^ Given their complementary yet opposing biological roles, integrating UA and HDL-C may better capture overall cardiometabolic imbalance. The UA to HDL-C ratio (UHR) has therefore been proposed as a composite biomarker capturing the joint information of UA-related metabolic burden and HDL-C-related lipid protection. In population-based studies, elevated UHR has been associated with a higher risk of adverse outcomes, such as increased all-cause and cardiovascular mortality and a greater likelihood of diabetic nephropathy.^[6,7]^ These observations suggest that UHR may capture cardiometabolic vulnerability beyond single conditions; however, whether UHR predicts the development of CMM, characterized by the coexistence of multiple CMDs, remains unclear.

Accordingly, we used data from the China Health and Retirement Longitudinal Study (CHARLS), a nationally representative longitudinal cohort of Chinese adults aged 45 years and older, to examine the prospective associations of baseline and cumulative UHR (CumUHR) levels with CMM. Elucidating the relationship between UHR and CMM may provide evidence for the use of a simple composite biomarker to characterize long-term cardiometabolic burden and improve risk stratification for CMM.

## 2. Method

### 2.1. Sample and study design

This study used data from CHARLS, a nationally representative longitudinal cohort of Chinese adults aged 45 years and older. CHARLS employs a multistage, stratified probability sampling design and includes detailed information on sociodemographic characteristics, health behaviors, physician-diagnosed chronic diseases, physical measurements, and fasting blood biomarkers. Wave 1 (2011–2012) served as the baseline, with follow-up surveys conducted in Waves 2 (2013), 3 (2015), and 4 (2018). The CHARLS protocol was approved by the Biomedical Ethics Review Committee of Peking University (IRB00001052-11015), and all participants provided written informed consent.^[8]^

**Figure 1.**
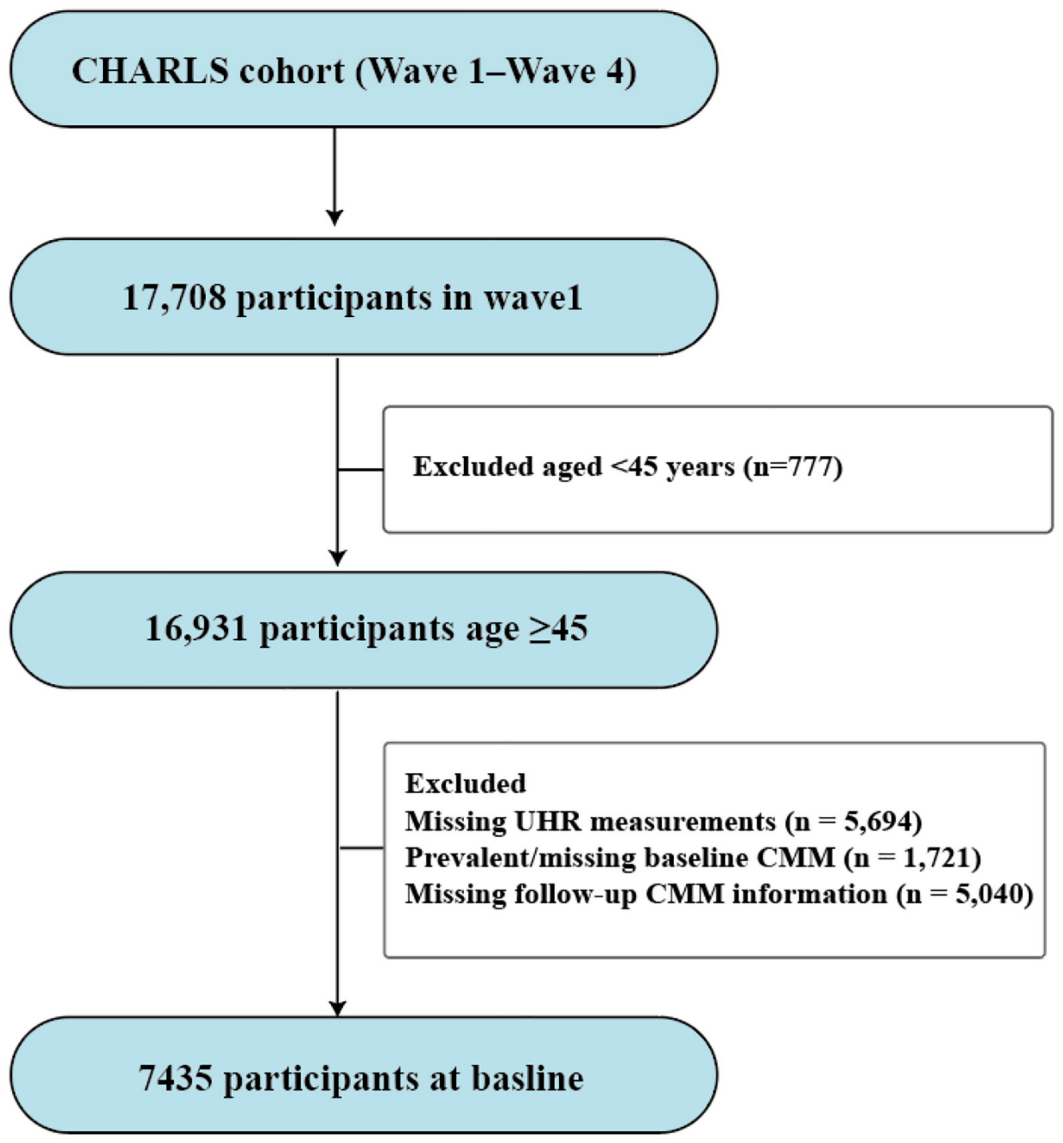
Flowchart of the study population. The flowchart shows the participant selection process from the CHARLS cohort and the exclusion criteria used to define the final analytic cohort for the baseline UHR analysis. CHARLS = China Health and Retirement Longitudinal Study, CMM = cardiometabolic multimorbidity, n = number of participants, UHR = uric acid-to-high-density lipoprotein cholesterol ratio.

To investigate the associations of baseline UHR and CumUHR with CMM, several exclusion criteria were sequentially applied. First, participants with missing UHR measurements required for the assessment of the exposure variables were excluded (n = 5694). Second, participants with prevalent CMM at baseline or missing baseline CMM information were excluded (n = 1721). Third, individuals without ascertainable CMM status during follow-up were excluded (n = 5040). After these exclusions, 7435 participants remained in the analytic cohort for the baseline UHR analysis. The detailed participant selection process for the baseline UHR analysis is illustrated in Figure 1. Baseline CMM status was determined using information collected in Wave 1 to exclude prevalent cases. CMM occurring during follow-up was identified in Waves 2, 3, or 4. Follow-up time was calculated from baseline to the first occurrence of CMM or the last available follow-up interview. For the CumUHR analysis, participants who had developed CMM by Wave 2 or Wave 3 were further excluded (n = 730), because CumUHR was calculated using UHR measurements from Waves 1 and 3. Accordingly, 6705 participants who remained free of CMM through 2015 were included in the CumUHR analysis.

### 2.2. Definition of UHR and CumUHR

In this study, UHR was calculated as the ratio of UA (mg/dL) to HDL-C (mg/dL). Both biomarkers were measured from fasting venous blood samples collected during the CHARLS survey using standardized laboratory procedures. CumUHR was estimated based on repeated measurements obtained in 2011 and 2015 using a time-weighted average approach: CumUHR = [(UHR2011 + UHR2015)/ 2] × time interval (2015−2011). The calculation methods were consistent with previous studies.^[9,10]^

### 2.3. Definition of CMM

Similar to prior investigations,^[2,11]^ CMM was defined as the coexistence of 2 or more CMDs, including heart disease, diabetes, stroke, and hypertension. The presence of CMDs was ascertained using standardized CHARLS questionnaire information and available physical examination or biochemical measurements. Hypertension was identified by self-reported physician diagnosis of hypertension or measured blood pressure levels, defined as systolic blood pressure ≥ 140 mm Hg and/or diastolic blood pressure ≥ 90 mm Hg. Diabetes was identified by self-reported physician diagnosis of diabetes or elevated blood glucose, fasting blood glucose ≥ 7.0 mmol/L, or hemoglobin A1c ≥ 6.5%. Heart disease was defined according to self-reported physician diagnosis of heart attack, coronary heart disease, angina, congestive heart failure, or other heart problems. Stroke was defined according to self-reported physician diagnosis of stroke, including cerebral infarction and cerebral hemorrhage. Incident CMM was defined as the occurrence of 2 or more CMDs during follow-up among participants without CMM at baseline.

### 2.4. Covariates

Covariates were selected based on prior literature and the availability of variables in the CHARLS database.^[12,13]^ Demographic factors included age, sex, education level, marital status, and residence (urban or rural). Lifestyle factors included smoking status and alcohol consumption. Body mass index (BMI) was included as an anthropometric indicator. Clinical and biochemical variables included estimated glomerular filtration rate (eGFR) and C-reactive protein (CRP), with eGFR calculated using the 2021 Chronic Kidney Disease Epidemiology Collaboration creatinine equation and expressed as mL/min/1.73 m^2^.^[14]^

### 2.5. Statistical analysis

For baseline variables with missing values other than those essential for defining UHR and CMM, multiple imputation was performed to reduce potential bias and loss of statistical power associated with complete-case analysis. Details of the missing values and imputed variables are provided in Table S1, Supplemental Digital Content 1. Baseline characteristics were summarized according to quartiles of UHR. The distributional characteristics of continuous variables were reassessed before analysis using the Shapiro–Wilk test and skewness. Approximately normally distributed continuous variables were presented as means with standard deviations, whereas skewed continuous variables were presented as medians with interquartile ranges. Categorical variables were presented as numbers and percentages. Differences across groups were compared using 1-way analysis of variance for approximately normally distributed continuous variables, the Kruskal–Wallis test for skewed continuous variables, and the *χ*^2^ test for categorical variables.

Time-to-event analyses were performed using Cox proportional hazards regression models to estimate hazard ratios (HRs) and 95% confidence intervals (CIs) for CMM. Follow-up time was calculated from baseline (2011) to the first occurrence of CMM or the last available follow-up interview. UHR was primarily analyzed in quartiles with the lowest quartile as the reference group, and linear trends were assessed by modeling quartile categories as an ordinal variable. UHR was also analyzed as a median-based dichotomous variable in sensitivity analyses. CumUHR was used to reflect long-term exposure burden. The optimal cut point for CumUHR was determined using maximally selected rank statistics, and the association between CumUHR and CMM during follow-up was further evaluated using Cox models and Kaplan–Meier curves with log-rank tests.

Three progressively adjusted Cox proportional hazards models were constructed. Model 1 was unadjusted. Model 2 adjusted for sociodemographic factors including age, sex, education level, marital status, and residence (urban/rural). Model 3 was further adjusted for smoking status, alcohol consumption, BMI, eGFR, and CRP. Restricted cubic spline analyses were conducted to explore potential nonlinear associations between UHR and the risk of CMM. Receiver operating characteristic (ROC) curve analyses were performed to compare the predictive performance of UHR and CumUHR with that of their individual components (UA and HDL-C).

All statistical analyses were performed using R software (version 4.3.1; R Foundation for Statistical Computing, Vienna, Austria). A 2-sided *P* value < .05 was considered statistically significant.

## 3. Results

### 3.1. Baseline characteristics of participants

A total of 7435 participants were included in the present analysis. During a median follow-up of approximately 7 years, 1748 participants (23.51%) developed CMM. Baseline characteristics stratified by CMM status are presented in Table 1. Participants who developed CMM were older and had a less favorable cardiometabolic profile at baseline compared with those without CMM (all *P* < .05). They also had higher baseline prevalences of hypertension, diabetes, heart disease, and stroke (all *P* < .01).

**Table 1:** Baseline characteristics of participants by CMM status.

Variables	Total	No. CMM	CMM	*P* value
N	7435	5687	1748	
Age, Yrs	58.26 (8.84)	57.63 (8.74)	60.32 (8.84)	< .001
Gender, n (%)				.002
Male	3424 (46.1)	2676 (47.1)	748 (42.8)	
Female	4011 (53.9)	3011 (52.9)	1000 (57.2)	
Marital status, n (%)				< .001
Married	6677 (89.8)	5167 (90.9)	1510 (86.4)	
Other	758 (10.2)	520 (9.1)	238 (13.6)	
Education level, n (%)				.013
Elementary school and below	5175 (69.6)	3916 (68.9)	1259 (72.0)	
Secondary school and above	2260 (30.4)	1771 (31.1)	489 (28.0)	
Residence, n (%)				.018
Rural	4981 (67.0)	3851 (67.7)	1130 (64.6)	
Urban	2454 (33.0)	1836 (32.3)	618 (35.4)	
Smoking Status, n (%)				.013
No	4574 (61.5)	3454 (60.7)	1120 (64.1)	
Yes	2861 (38.5)	2233 (39.3)	628 (35.9)	
Drinking Status, n (%)				.25
No	4547 (61.2)	3457 (60.8)	1090 (62.4)	
Yes	2888 (38.8)	2230 (39.2)	658 (37.6)	
BMI, kg/m^2^	23.54 (3.91)	23.09 (3.71)	25.00 (4.19)	< .001
FBG, mg/dL	101.88 [94.14, 112.14]	100.98 [93.60, 109.80]	106.02 [96.48, 121.14]	< .001
HbA1c, %	5.10 [4.90, 5.40]	5.10 [4.80, 5.40]	5.20 [4.90, 5.60]	< .001
SBP, mm Hg	128.06 (20.69)	125.13 (19.54)	137.61 (21.48)	< .001
DBP, mm Hg	74.90 (12.04)	73.55 (11.61)	79.28 (12.36)	< .001
TC, mg/dL	193.31 (38.20)	192.42 (37.90)	196.23 (39.03)	< .001
TG, mg/dL	104.43 [74.34, 153.10]	100.00 [72.57, 145.14]	123.01 [84.07, 175.23]	< .001
HDL-C, mg/dL	51.49 (15.41)	52.35 (15.53)	48.67 (14.67)	< .001
LDL-C, mg/dL	116.15 (34.60)	115.52 (33.79)	118.20 (37.05)	.003
UA, mg/dL	4.40 (1.21)	4.37 (1.18)	4.50 (1.28)	< .001
eGFR, ml/min/1.73m^2^	93.11 (14.19)	93.87 (13.89)	90.62 (14.84)	< .001
CRP, mg/L	0.97 [0.53, 2.02]	0.90 [0.50, 1.86]	1.26 [0.65, 2.61]	< .001
Dyslipidaemia, n (%)				< .001
No	6870 (92.4)	5371 (94.4)	1499 (85.8)	
Yes	565 (7.6)	316 (5.6)	249 (14.2)	
LL medication, n (%)				
No	7158 (96.3)	5536 (97.3)	1622 (92.8)	< .001
Yes	277 (3.7)	151 (2.7)	126 (7.2)	
Hypertension, n (%)				< .001
No	5914 (79.5)	4885 (85.9)	1029 (58.9)	
Yes	1521 (20.5)	802 (14.1)	719 (41.1)	
Diabetes, n (%)				< .001
No	7221 (97.1)	5590 (98.3)	1631 (93.3)	
Yes	214 (2.9)	97 (1.7)	117 (6.7)	
Heart disease, n (%)				
No	6978 (93.9)	5452 (95.9)	1526 (87.3)	< .001
Yes	457 (6.1)	235 (4.1)	222 (12.7)	
Stroke, n (%)				
No	7372 (99.2)	5653 (99.4)	1719 (98.3)	< .001
Yes	63 (0.8)	34 (0.6)	29 (1.7)	

Continuous variables are presented as mean ± SD or median (IQR), as appropriate, and categorical variables are presented as n (%). *P* values were calculated using 1-way analysis of variance for approximately normally distributed continuous variables, the Kruskal–Wallis test for skewed continuous variables, and the *χ*^2^ test for categorical variables.

BMI = body mass index, CMM = cardiometabolic multimorbidity, CRP = C-reactive protein, DBP = diastolic blood pressure, eGFR = estimated glomerular filtration rate, FBG = fasting blood glucose, HbA1c = hemoglobin A1c, HDL-C = high-density lipoprotein cholesterol, IQR = interquartile range, LDL-C = low-density lipoprotein cholesterol, LL medication = lipid-lowering medication, N/n = number of participants, SBP = systolic blood pressure, SD = standard deviation, TC = total cholesterol, TG = triglycerides, UA = uric acid, UHR = uric acid-to-high-density lipoprotein cholesterol ratio.

The baseline characteristics of the study population according to quartiles of UHR are shown in Table 2 (Q1 ≤ 6.39; Q2 6.39–8.49; Q3 8.49–11.42; Q4 > 11.42). Participants in the highest UHR quartile were slightly older than those in the lower quartiles (*P* = .034), and the proportion of men increased progressively across quartiles (*P* < .001). Higher UHR quartiles were also associated with higher levels of BMI, fasting blood glucose, systolic and diastolic blood pressure, triglycerides, serum UA, and CRP, as well as lower HDL-C concentrations (all *P* < .001). In addition, current smoking and alcohol consumption were more frequent in the higher UHR quartiles. The prevalences of hypertension, diabetes, and heart disease differed significantly across UHR quartiles (all *P* < .001), while the prevalence of stroke showed a slight increasing tendency with higher UHR quartiles but did not reach statistical significance, possibly due to the small number of stroke cases at baseline (*P* = .655).

**Table 2:** Baseline characteristics of study participants according to quartiles of UHR.

Variables	Total	Quartiles of UHR				*P* value
		Q1	Q2	Q3	Q4	
N	7435	1859	1858	1859	1859	
Age, Yrs	58.26 (8.84)	57.70 (8.48)	58.29 (9.05)	58.47 (8.88)	58.57 (8.91)	.013
Gender, n (%)						< .001
Male	3424 (46.1)	520 (28.0)	750 (40.4)	981 (52.8)	1173 (63.1)	< .001
Female	4011 (53.9)	1339 (72.0)	1108 (59.6)	878 (47.2)	686 (36.9)	
Marital status, n (%)						.01
Married	6677 (89.8)	1638 (88.1)	1660 (89.3)	1695 (91.2)	1684 (90.6)	
Other	758 (10.2)	221 (11.9)	198 (10.7)	164 (8.8)	175 (9.4)	
Education level, n (%)						< .001
Elementary school and below	5175 (69.6)	1391 (74.8)	1336 (71.9)	1261 (67.8)	1187 (63.9)	
Secondary school and above	2260 (30.4)	468 (25.2)	522 (28.1)	598 (32.2)	672 (36.1)	
Residence, n (%)						< .001
Rural	4981 (67.0)	1388 (74.7)	1280 (68.9)	1226 (65.9)	1087 (58.5)	
Urban	2454 (33.0)	471 (25.3)	578 (31.1)	633 (34.1)	772 (41.5)	
Smoking Status, n (%)						< .001
No	4574 (61.5)	1374 (73.9)	1205 (64.9)	1064 (57.2)	931 (50.1)	< .001
Yes	2861 (38.5)	485 (26.1)	653 (35.1)	795 (42.8)	928 (49.9)	
Drinking Status, n (%)						
No	4547 (61.2)	1276 (68.6)	1200 (64.6)	1085 (58.4)	986 (53.0)	< .001
Yes	2888 (38.8)	583 (31.4)	658 (35.4)	774 (41.6)	873 (47.0)	
BMI, kg/m^2^	23.54 (3.91)	22.36 (3.37)	23.04 (3.87)	23.89 (3.93)	24.87 (3.99)	< .001
FBG, mg/dL	101.88 [94.14, 112.14]	100.08 [92.88, 108.99]	101.16 [93.96, 109.62]	102.06 [93.96, 112.95]	105.12 [96.48, 118.80]	< .001
HbA1c, %	5.10 [4.90, 5.40]	5.10 [4.90, 5.40]	5.10 [4.90, 5.40]	5.10 [4.90, 5.40]	5.20 [4.90, 5.50]	< .001
SBP, mm Hg	128.06 (20.69)	124.69 (19.52)	126.84 (20.81)	129.15 (20.92)	131.56 (20.88)	< .001
DBP, mm Hg	74.90 (12.04)	72.90 (11.49)	73.83 (11.92)	75.56 (12.05)	77.30 (12.23)	< .001
TC, mg/dL	193.31 (38.20)	198.62 (36.33)	192.53 (35.03)	191.20 (37.94)	190.89 (42.59)	< .001
TG, mg/dL	104.43 [74.34, 153.10]	79.65 [61.06, 107.08]	94.69 [69.92, 130.10]	111.51 [81.42, 158.41]	153.99 [105.76, 237.18]	< .001
HDL-C, mg/dL	51.49 (15.41)	66.59 (15.08)	54.90 (10.36)	47.22 (8.94)	37.24 (8.64)	< .001
LDL-C, mg/dL	116.15 (34.60)	117.42 (32.45)	117.94 (31.76)	118.88 (33.77)	110.38 (39.29)	< .001
UA, mg/dL	4.40 (1.21)	3.36 (0.76)	4.07 (0.76)	4.62 (0.88)	5.54 (1.16)	< .001
eGFR, ml/min/1.73m^2^	93.11 (14.19)	97.43 (11.54)	94.12 (13.21)	92.29 (14.22)	88.59 (15.95)	<.001
CRP, mg/L	0.97 [0.53, 2.02]	0.67 [0.41, 1.28]	0.89 [0.49, 1.86]	1.06 [0.58, 2.13]	1.43 [0.75, 2.84]	< .001
Dyslipidaemia, n (%)						< .001
No	6870 (92.4)	1771 (95.3)	1730 (93.1)	1711 (92.0)	1658 (89.2)	
Yes	565 (7.6)	88 (4.7)	128 (6.9)	148 (8.0)	201 (10.8)	
LL medication, n (%)						
No	7158 (96.3)	1816 (97.7)	1797 (96.7)	1789 (96.2)	1756 (94.5)	< .001
Yes	277 (3.7)	43 (2.3)	61 (3.3)	70 (3.8)	103 (5.5)	
Hypertension, n (%)						< .001
No	5914 (79.5)	1596 (85.9)	1540 (82.9)	1441 (77.5)	1337 (71.9)	
Yes	1521 (20.5)	263 (14.1)	318 (17.1)	418 (22.5)	522 (28.1)	
Diabetes, n (%)						< .001
No	7221 (97.1)	1812 (97.5)	1806 (97.1)	1803 (97.0)	1800 (96.8)	
Yes	214 (2.9)	46 (2.5)	53 (2.9)	56 (3.0)	59 (3.2)	
Heart disease, n (%)						
No	6978 (93.9)	1756 (94.5)	1748 (94.0)	1745 (93.9)	1729 (93.0)	< .001
Yes	457 (6.1)	102 (5.5)	111 (6.0)	114 (6.1)	130 (7.0)	
Stroke, n (%)						
No	7372 (99.2)	1844 (99.2)	1845 (99.2)	1842 (99.1)	1841 (99.1)	.655
Yes	63 (0.8)	15 (0.8)	14 (0.8)	17 (0.9)	17 (0.9)	

Continuous variables are presented as mean ±  SD or median (IQR), as appropriate, and categorical variables are presented as n (%). *P* values were calculated using 1-way analysis of variance for approximately normally distributed continuous variables, the Kruskal–Wallis test for skewed continuous variables, and the *χ*^2^ test for categorical variables.

BMI = body mass index, CMM = cardiometabolic multimorbidity, CRP = C-reactive protein, DBP = diastolic blood pressure, eGFR = estimated glomerular filtration rate, FBG = fasting blood glucose, HbA1c = hemoglobin A1c, HDL-C = high-density lipoprotein cholesterol, IQR = interquartile range, LDL-C = low-density lipoprotein cholesterol, LL medication = lipid-lowering medication, N/n = number of participants, SBP = systolic blood pressure, SD = standard deviation, TC = total cholesterol, TG = triglycerides, UA = uric acid, UHR = uric acid-to-high-density lipoprotein cholesterol ratio.

### 3.2. Association between UHR and CMM

**Figure 2.**
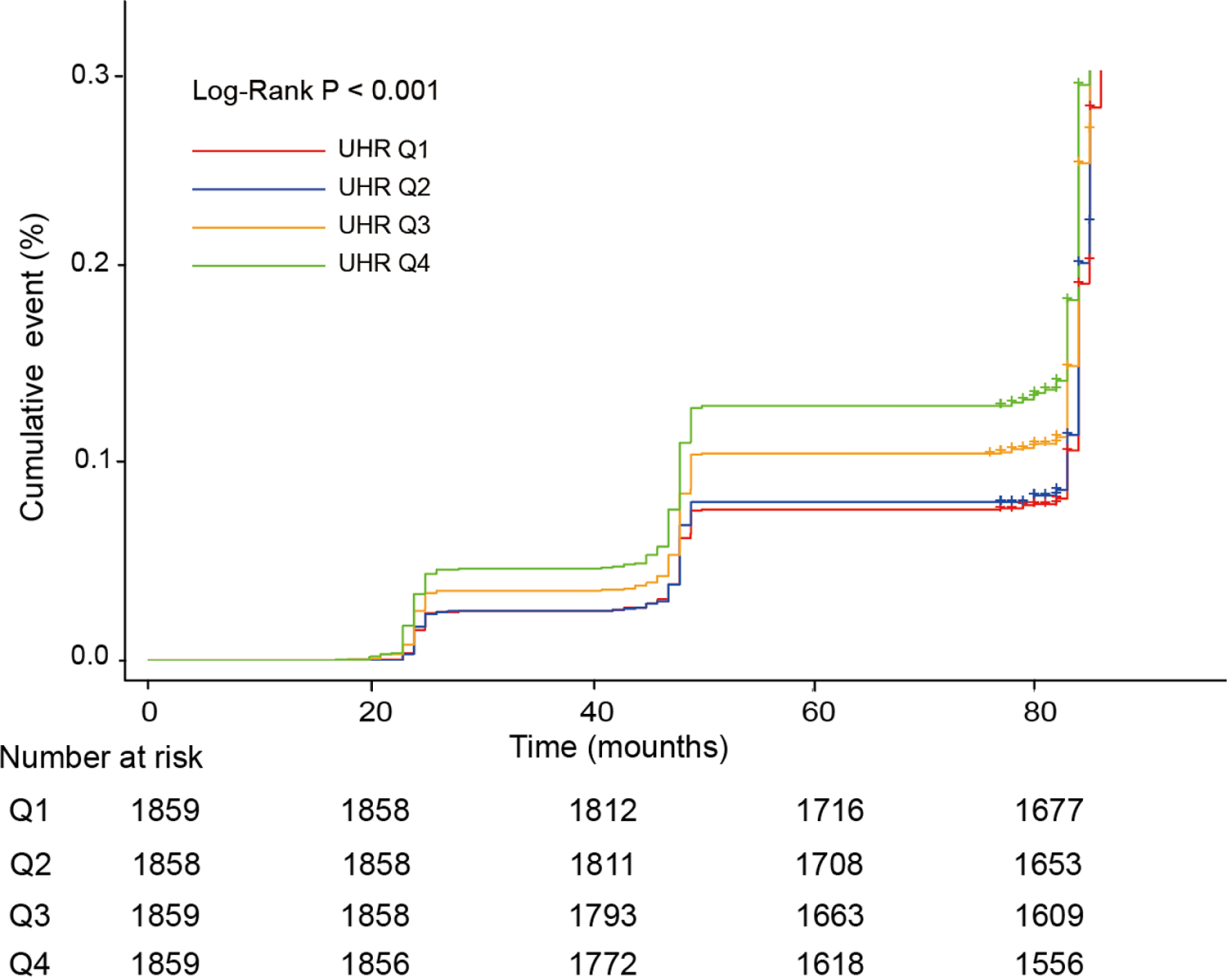
Kaplan–Meier curves for cumulative events of CMM across UHR quartiles. Participants were divided into quartiles according to baseline UHR levels. The cumulative event rate of CMM was compared across UHR quartiles. The number at risk at each time point is shown below the plot. CMM = cardiometabolic multimorbidity, UHR = uric acid-to-high-density lipoprotein cholesterol ratio.

Associations between UHR and incident CMM across Cox proportional hazards models are presented in Table 3. Model 1 was unadjusted, Model 2 adjusted for sociodemographic factors, and Model 3 further adjusted for lifestyle and metabolic factors. In the fully adjusted model, participants in the highest quartile of UHR had a significantly higher risk of CMM than those in the lowest quartile (HR 1.48, 95% CI 1.27–1.77; *P* for trend = .007). Similar associations were observed in the unadjusted and partially adjusted models. Kaplan–Meier curves showed progressively higher cumulative event rates of CMM across increasing quartiles of UHR (Fig. 2; log-rank *P* < .001).

**Table 3:** Association between UHR and CMM.

UHR quartile	Model 1		Model 2		Model 3	
	HR (95% CI)	*P* value	HR (95% CI)	*P* value	HR (95% CI)	*P* value
Q1	Reference		Reference		Reference	
Q2	1.10 (0.95–1.27)	.196	1.13 (0.97–1.30)	.108	0.98 (0.84–1.13)	.743
Q3	1.40 (1.22–1.60)	< .001	1.50 (1.30–1.73)	< .001	1.09 (0.94–1.25)	.258
Q4	1.93 (1.68–2.22)	< .001	1.76 (1.54–2.01)	< .001	1.48 (1.27–1.77)	.015
*P* for trend		< .001		< .001		.007

Model 1 was unadjusted. Model 2 was adjusted for age, sex, education level, marital status, and residence. Model 3 was further adjusted for smoking status, alcohol consumption, BMI, estimated glomerular filtration rate, and CRP.

BMI = body mass index, CI = confidence interval, CMM = cardiometabolic multimorbidity, CRP = C-reactive protein, HR = hazard ratio, UHR = uric acid-to-high-density lipoprotein cholesterol ratio.

### 3.3. Association between CumUHR and CMM

For the CumUHR analysis, 730 participants who had developed CMM by 2013 or 2015 were excluded to ensure that CumUHR, calculated using UHR measurements from 2011 and 2015, preceded the subsequent outcome assessment. A total of 6705 participants remained for the CumUHR analysis, among whom 1018 developed incident CMM by 2018 and 5687 remained free of CMM. The optimal cutoff value of CumUHR for predicting CMM was 41.59 (Fig. S1, Supplemental Digital Content 2). Participants were categorized into low (< 41.59) and high (≥ 41.59) CumUHR groups. Higher CumUHR was associated with a greater risk of CMM (Table 4). In the fully adjusted model, participants with high CumUHR had a significantly higher risk of CMM than those with low CumUHR (HR 1.26, 95% CI 1.15–1.39; *P* = .004). Similar associations were observed in the unadjusted and partially adjusted models.

**Table 4:** Association between CumUHR and CMM.

CumUHR	Model 1		Model 2		Model 3	
	HR (95% CI)	*P* value	HR (95% CI)	*P* value	HR (95% CI)	*P* value
Low	Reference		Reference		Reference	
High	1.52 (1.38–1.67)	< .001	1.63 (1.47–1.79)	<.001	1.26 (1.15–1.39)	.004
*P* value		< .001		<.001		.004

Model 1 was unadjusted. Model 2 was adjusted for age, sex, education level, marital status, and residence. Model 3 was further adjusted for smoking status, alcohol consumption, BMI, estimated glomerular filtration rate, and CRP.

BMI = body mass index, CI = confidence interval, CMM = cardiometabolic multimorbidity, CRP = C-reactive protein, CumUHR = cumulative UHR, HR = hazard ratio, UHR = uric acid-to-high-density lipoprotein cholesterol ratio.

### 3.4. Dose-response associations of UHR and CumUHR with CMM

**Figure 3.**
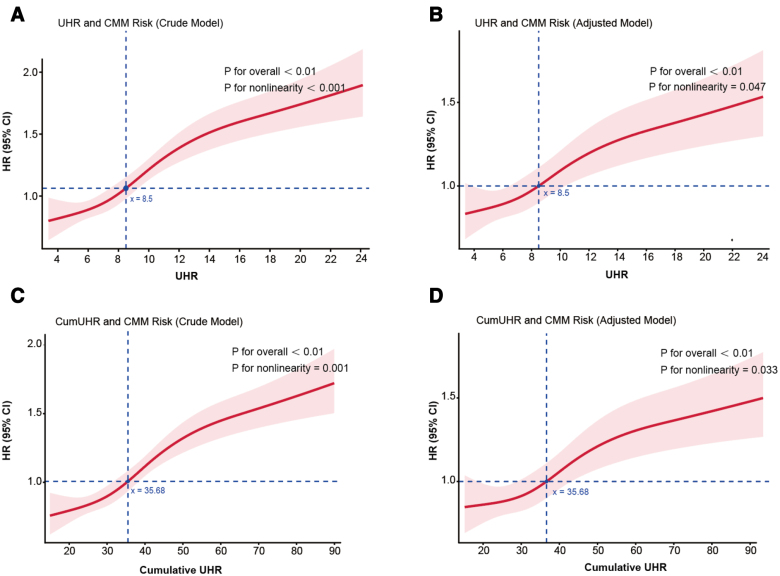
Restricted cubic spline analyses of the associations of UHR and CumUHR with the risk of CMM. Panels (A) and (B) show the associations between baseline UHR and CMM in the crude and multivariable-adjusted models, respectively. Panels (C) and (D) show the associations between CumUHR and CMM in the crude and multivariable-adjusted models, respectively. Solid lines represent hazard ratios, and shaded areas represent 95% confidence intervals. The horizontal dashed line indicates HR = 1.0, and the vertical dashed line indicates the reference value. *P* values for overall association and nonlinearity were obtained from the restricted cubic spline models. CI = confidence interval, CMM = cardiometabolic multimorbidity, CumUHR = cumulative uric acid-to-high-density lipoprotein cholesterol ratio; HR = hazard ratio; UHR uric acid-to-high-density lipoprotein cholesterol ratio.

Restricted cubic spline analyses were performed to evaluate the dose-response associations of UHR and CumUHR with CMM (Fig. 3). For UHR, significant overall associations with CMM were observed in both the crude and adjusted models (both *P* for overall < .01), and the adjusted model showed a significant nonlinear association (*P* for nonlinearity = .047), with an inflection point around 8.5. For CumUHR, significant overall associations were also observed in both the crude and adjusted models (both *P* for overall < .01), with significant nonlinearity in both the crude model (*P* for nonlinearity = .001) and the adjusted model (*P* for nonlinearity = .033), and an inflection point around 35.7. In both analyses, the risk of CMM increased with increasing levels of UHR and CumUHR.

### 3.5. Predictive performance of UHR and CumUHR for CMM

**Figure 4.**
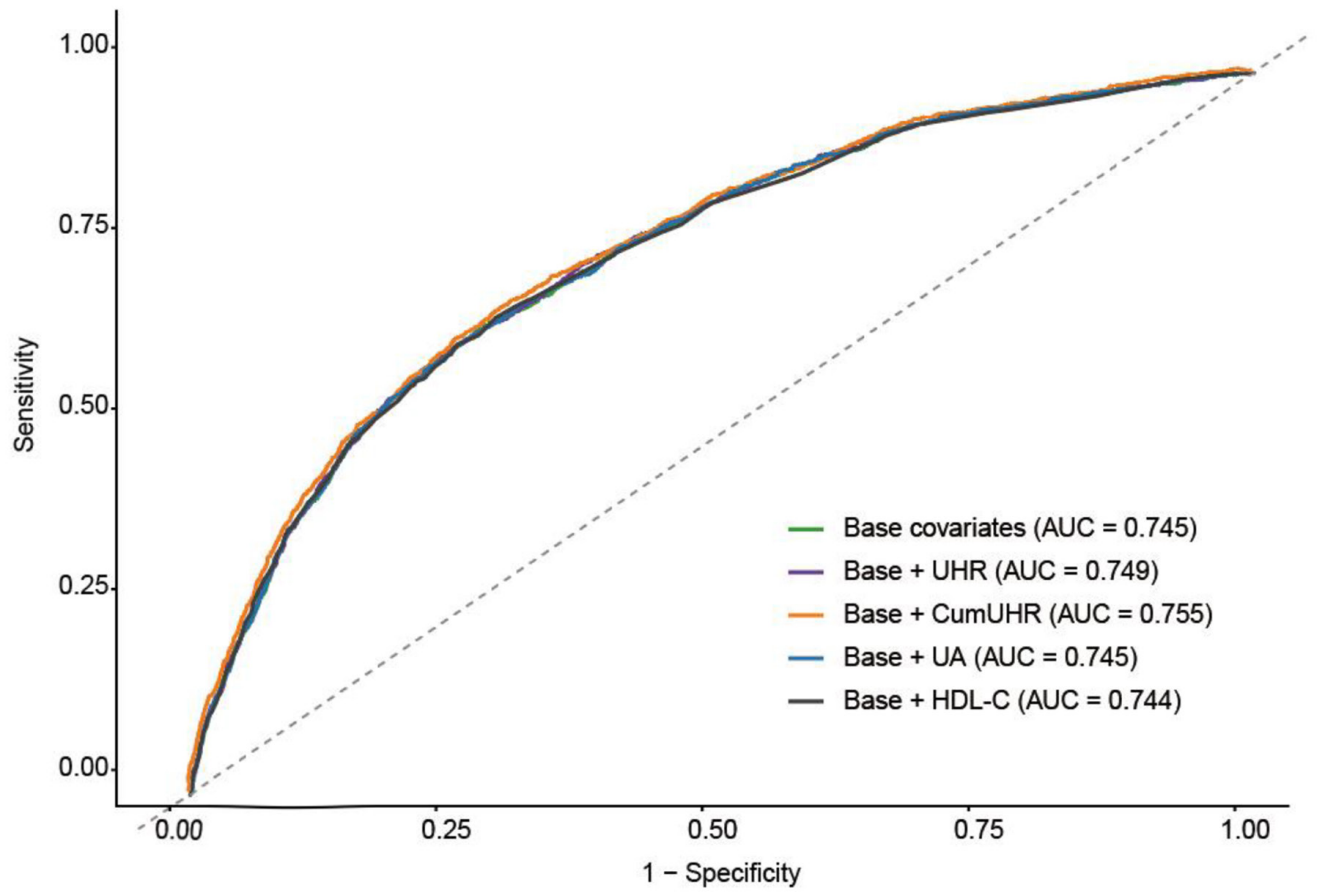
Predictive performance of UHR and CumUHR for CMM. ROC curves were used to compare the predictive performance of the base model and models additionally including UHR, CumUHR, UA, or HDL-C for incident CMM. The AUCs values are shown in the legend. The diagonal dashed line represents the reference line for no discrimination. AUC = area under the curve, CMM = cardiometabolic multimorbidity, CumUHR = cumulative uric acid-to-high-density lipoprotein cholesterol ratio, HDL-C = high-density lipoprotein cholesterol, ROC = receiver operating characteristic, UA = uric acid, UHR = uric acid-to-high-density lipoprotein cholesterol ratio.

ROC curve results are presented in Table 5 and Figure 4. The base model yielded an area under the curve (AUC) of 0.745 (95% CI 0.732–0.758). After adding UHR, which integrates UA and HDL-C, the AUC increased to 0.749 (95% CI 0.735–0.761; *P* = .041), whereas the model including CumUHR yielded an AUC of 0.755 (95% CI 0.745–0.761; *P* = .028). For the individual components of UHR, the corresponding AUCs were 0.745 (95% CI 0.732–0.758; *P* = .833) for UA and 0.744 (95% CI 0.734–0.759; *P* = .833) for HDL-C. In addition, UHR and CumUHR showed statistically significant improvements in both net reclassification improvement and integrated discrimination improvement, with slightly greater incremental improvement observed for CumUHR. In contrast, the addition of UA or HDL-C alone did not significantly improve AUC, net reclassification improvement, or integrated discrimination improvement.

**Table 5:** Predictive performance of UHR and CumUHR for CMM.

Model	AUC (95% CI)	*P* value	NRI (95% CI)	*P* value	IDI (95% CI)	*P* value
Base model	0.745 (0.732–0.758)	Ref	Ref		Ref	
+ UHR	0.749 (0.735–0.761)	.041	0.052 (0.012–0.103)	.037	0.004 (0.001–0.005)	.006
+ CumUHR	0.755 (0.745–0.761)	.028	0.063 (0.013–0.112)	.013	0.007 (0.003–0.009)	.001
+ UA	0.745 (0.732–0.758)	.833	0.014 (−0.036–0.064)	.578	0.003 (−0.001–0.005)	.112
+ HDL-C	0.744 (0.734–0.759)	.833	0.017 (−0.005–0.047)	.115	0.002 (−0.001–0.006)	.217

The base model was adjusted for age, sex, education level, marital status, residence, smoking status, alcohol consumption, body mass index, estimated glomerular filtration rate, and C-reactive protein.

AUC = area under the curve, CI = confidence interval, CMM = cardiometabolic multimorbidity, CumUHR = cumulative UHR, HDL-C = high-density lipoprotein cholesterol, IDI = integrated discrimination improvement, NRI = net reclassification improvement, UA = uric acid, UHR = uric acid-to-high-density lipoprotein cholesterol ratio.

### 3.6. Subgroup and sensitivity analyses

**Figure 5.**
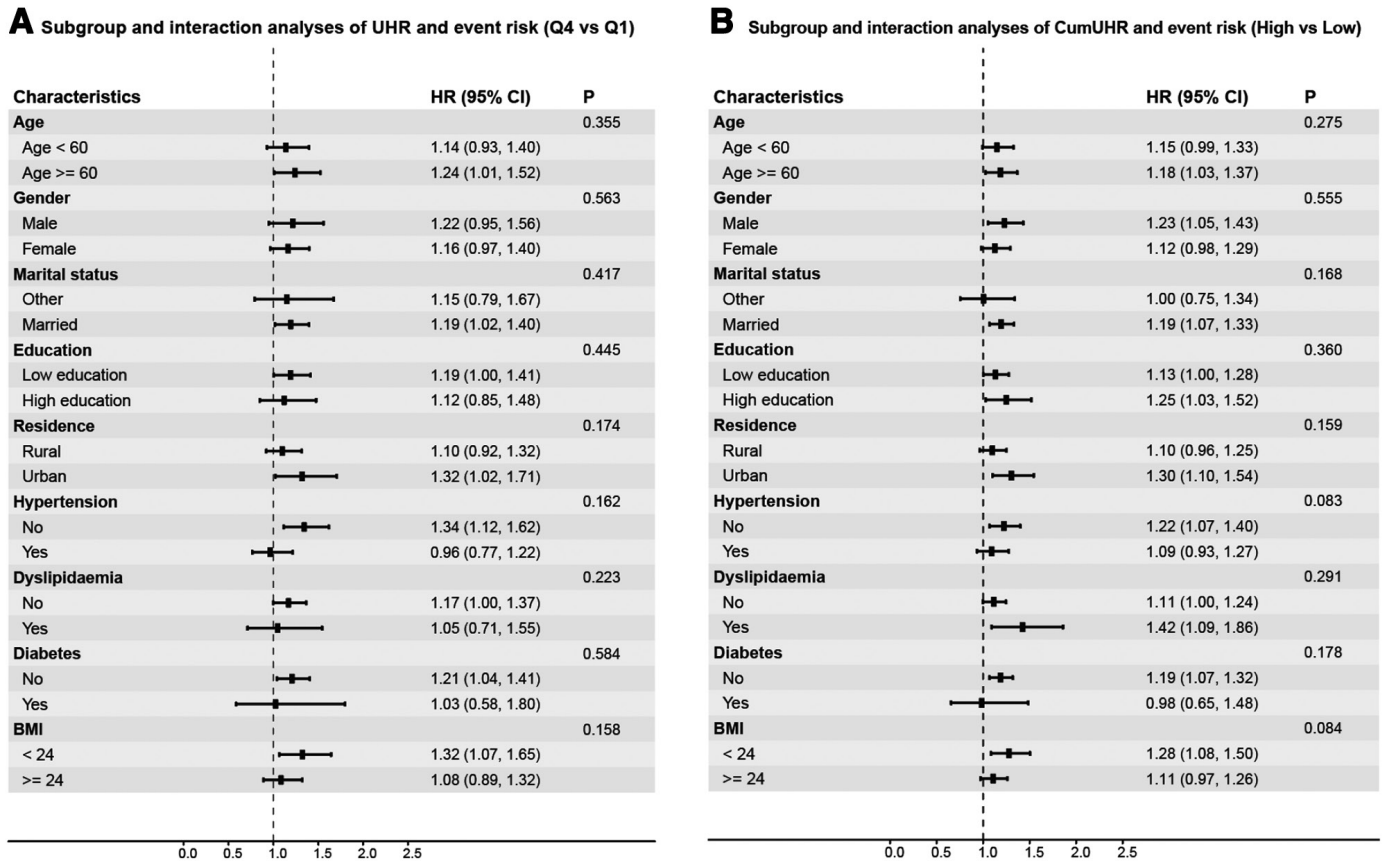
Subgroup and interaction analyses of baseline UHR and cumulative UHR in relation to CMM. Panel (A) shows HRs for incident CMM comparing the highest versus lowest quartile of baseline UHR (Q4 vs Q1) across predefined subgroups. Panel (B) shows HRs comparing high versus low cumulative UHR. HRs were estimated using multivariable Cox proportional hazards models adjusted for the same covariates as in the primary analysis. Squares indicate point estimates, and horizontal lines indicate 95% CIs. The vertical dashed line denotes HR = 1.0. *P* values represent tests for interaction between esxposure and subgroup variable. BMI = body mass index, CI = confidence interval, CMM = cardiometabolic multimorbidity, CumUHR = cumulative uric acid-to-high-density lipoprotein cholesterol ratio, HR = hazard ratio, UHR = uric acid-to-high-density lipoprotein cholesterol ratio.

The associations of UHR and CumUHR with CMM were broadly consistent across most predefined subgroups, and no significant interactions were observed for either UHR or CumUHR (all *P* for interaction > .05; Fig. 5).

Sensitivity analyses based on the median value of baseline UHR were conducted (Table S2, Supplemental Digital Content 3). Participants with baseline UHR ≥ median had a significantly higher risk of CMM than those with baseline UHR < median (adjusted HR 1.16, 95% CI 1.05–1.29; *P* = .004). Kaplan–Meier curves also showed higher cumulative event rates of CMM among participants with higher baseline UHR levels (log-rank *P* < .001; Figure S2, Supplemental Digital Content 4). Subgroup analyses showed no significant interactions between baseline UHR (≥ median vs < median) and the examined variables (all *P* for interaction > .05; Figure S3, Supplemental Digital Content 5).

In addition, sensitivity analyses with additional adjustment for lipid-lowering drug use were performed. The associations of both UHR and CumUHR with CMM remained materially unchanged after further adjustment for lipid-lowering drug use in the fully adjusted Cox model (Table S3, Supplemental Digital Content 6 and Table S4, Supplemental Digital Content 7).

## 4. Discussion

In this prospective cohort study of middle-aged and older Chinese adults, higher UHR levels were independently associated with an increased risk of CMM during follow-up. Similar findings were observed for CumUHR, indicating that both baseline UHR and cumulative exposure burden were relevant to CMM development, with a progressive increase in risk at higher levels. ROC analyses showed that UHR and CumUHR, which integrate information from UA and HDL-C, provided incremental predictive information beyond conventional cardiometabolic risk factors. These findings support the potential role of UHR as a simple and easily obtainable complementary biomarker for cardiometabolic risk assessment, although its incremental discriminative improvement was modest and should be interpreted together with established clinical risk factors.

Several considerations may help explain why UHR is associated with the risk of CMM during follow-up. UHR integrates UA and HDL-C, 2 biomarkers with complementary cardiometabolic implications.^[15]^ Elevated UA has long been linked to a higher risk of CMDs such as hypertension and heart disease.^[16–18]^ A meta-analysis of 8 observational studies including 21,832 participants further reported that higher UA levels were associated with an increased risk of prehypertension.^[19]^ Mendelian randomization analyses have also supported a causal relationship between genetically predicted UA levels and CMDs.^[20]^ In contrast, HDL-C is generally regarded as protective for lipid metabolism and vascular health.^[21]^ As a composite indicator integrating adverse metabolic burden reflected by UA and vascular protection reflected by HDL-C, UHR may provide a more comprehensive assessment of cardiometabolic risk than either marker alone. Consistent with this concept, several studies have reported associations between UHR and cardiometabolic outcomes, such as CVD and diabetes.^[22,23]^ Furthermore, an National Health and Nutrition Examination Survey-based analysis showed that UHR achieved superior overall discriminatory ability for hypertension compared with its individual components, supporting the value of combining UA and HDL information into a composite indicator.^[24]^ In addition, our focus on CMM rather than individual CMDs is clinically meaningful, because CMDs rarely occur in isolation and tend to accumulate with age.^[25,26]^ Evidence from the China Kadoorie Biobank suggests that CMM commonly clusters as diabetes, coronary heart disease, stroke, and hypertension.^[2]^ Thus, compared with a single CMD outcome, CMM may better reflect the real-world clustering and accumulation of cardiometabolic abnormalities. Consistent with this concept, a recent prospective study reported that higher baseline UHR levels were associated with an increased risk of CMM.^[27]^ Our findings further extend these observations by additionally evaluating CumUHR based on repeated measurements. In the present study, ROC analyses showed that UHR provided additional predictive information for CMM compared with either UA or HDL-C alone, whereas CumUHR showed slightly greater incremental predictive value among the evaluated biomarkers. Several prospective analyses have also suggested that persistent or worsening adverse metabolic exposure over time is associated with a higher risk of CMM progression.^[28,29]^ Given that CMM develops through the progressive accumulation of CMDs over time,^[30]^ CumUHR may therefore provide additional prognostic information beyond a single baseline assessment by better capturing sustained metabolic burden.

Elevated UHR may reflect a biological milieu characterized by the coexistence of increased UA-related metabolic and inflammatory stress and reduced HDL-mediated vascular protection.^[31–33]^ Experimental and clinical evidence indicates that elevated UA promotes oxidative stress and inflammatory activation and contributes to endothelial dysfunction.^[34,35]^ Consistent with this inflammatory relevance, recent studies have shown that UA-related composite markers, such as the UA-to-albumin ratio, were associated with atrial fibrillation recurrence after catheter ablation and showed predictive value among inflammatory markers after cryoballoon ablation,^[36,37]^ providing supportive evidence linking UA-related biomarkers with inflammation-related cardiovascular outcomes. It has also been linked to activation of the renin–angiotensin–aldosterone system, insulin resistance, and other features of metabolic syndrome.^[38]^ At the same time, insulin resistance is increasingly recognized as a central pathophysiological hub in CMM,^[39]^ linking metabolic dysregulation with endothelial injury and β-cell failure.^[40]^ In this context, elevated UA may promote the progression of interconnected cardiometabolic abnormalities through shared pathways of oxidative stress, vascular dysfunction, and impaired glucose metabolism. In contrast, HDL exerts multiple protective effects beyond cholesterol transport.^[41]^ In addition, HDL-related indices have been used to reflect acute inflammatory and oxidative stress. The monocyte-to-HDL ratio was reported to be significantly higher in patients who developed postoperative atrial fibrillation after aortocoronary bypass graft surgery, supporting the link between reduced HDL-related anti-inflammatory capacity and inflammation-driven cardiovascular stress.^[42]^ HDL promotes endothelial nitric oxide production, helps preserve endothelial integrity, and possesses antioxidative and anti-inflammatory properties that may counteract vascular injury and atherogenesis.^[43]^ These protective functions are also relevant in diabetes and atherosclerotic CVD, where impaired HDL functionality has been linked to reduced antioxidant capacity, diminished anti-inflammatory effects, and altered nitric oxide signaling.^[44]^ Notably, UA- and HDL-C-related metabolic, inflammatory, and vascular pathways may also be involved in broader metabolic phenotypes that predispose individuals to CMM, including hepatic metabolic dysfunction. A recent meta-analysis showed that metabolic dysfunction-associated steatotic liver disease was associated with impaired left ventricular global longitudinal strain in individuals without overt heart disease,^[45]^ suggesting that future studies could explore whether UHR helps identify individuals with hepatic metabolic dysfunction who are at higher risk of CMM and early cardiac involvement. Taken together, a higher UHR may indicate that UA-related adverse metabolic signals outweigh HDL-associated vascular protection, thereby favoring the clustering and co-development of multiple cardiometabolic disorders rather than a single isolated disease.

This study has several strengths. First, this study used a large, nationally representative prospective cohort of middle-aged and older Chinese adults, supporting clear temporality between UHR and CMM and improving the generalizability of our findings within this population. Second, we evaluated both baseline UHR and CumUHR based on repeated measurements, allowing assessment of long-term exposure burden. Third, we assessed the clinical utility of UHR by comparing its discriminatory performance with UA and HDL-C using ROC analyses, providing interpretable evidence for the added value of this composite indicator in predicting CMM. Notably, CumUHR showed the highest discriminative performance among the evaluated biomarkers, highlighting the potential value of cumulative exposure assessment. Fourth, focusing on CMM rather than individual CMDs enhances clinical relevance by capturing the real-world clustering and accumulation of cardiometabolic conditions over time.

Several limitations should be noted. First, CHARLS contains some missing or unavailable information that may influence the interpretation of our findings. Although multiple imputation was used to address missing baseline variables, potential bias related to missing data could not be completely excluded. In addition, CHARLS lacks detailed information on diet, such as purine/fructose intake, urate-lowering therapy, and some cardiometabolic medications, which may influence UA/HDL-C levels and CMM risk.^[46,47]^ Therefore, although we adjusted for a range of demographic and clinical covariates, residual confounding could not be fully excluded. Second, although hypertension and diabetes were defined using both self-reported physician diagnoses and objective measurements, heart disease and stroke mainly relied on self-reported physician diagnoses in CHARLS. Therefore, recall bias, reporting bias, underdiagnosis, and potential disease misclassification cannot be completely excluded. Third, exposure measurement is limited. UHR and CumUHR were derived from measurements at only 2 time points (2011 and 2015), which may not fully capture short-term fluctuations or longer-term trajectories between and after visits. Future studies with more detailed covariate information, more frequent repeated biomarker assessments, and validated outcome ascertainment are warranted to confirm and extend these findings.

## 5. Conclusion

In this prospective cohort study of middle-aged and older Chinese adults, both baseline UHR and CumUHR were independently associated with a higher risk of CMM. As composite indicators integrating UA and HDL-C, UHR and CumUHR may provide complementary information for cardiometabolic risk assessment. These findings support the potential value of UHR and CumUHR as simple adjunctive markers for identifying individuals at elevated CMM risk when considered together with established clinical risk factors.

## Acknowledgments

The researchers express their gratitude to the CHARLS research team and all participants involved in this study.

## Author contributions

**Conceptualization:** Shijing Jiang.

**Data curation:** Shuliang Wang.

**Project administration:** Zhiwei Miao.














