# Supplementary material for: Predictive value of uric acid-to-high-density lipoprotein cholesterol ratio for cardiometabolic multimorbidity in middle-aged and older adults: A nationwide prospective cohort study
Source: Medicine (Baltimore). 2026 Jul 10;105(28):e49740. doi: 10.1097/MD.0000000000049740 (PMC13362854; doi:10.1097/MD.0000000000049740)
Supplement: Supplementary file 1 [file medi-105-e49740-s001.docx]

**TableS1. Missing values of variables included in the multiple imputation procedure**

| **Variable** | **Missing values, n** | **Missing proportion, %** |
| --- | --- | --- |
| **Smoking status** | 9 | 0.12 |
| **Drinking status** | 14 | 0.19 |
| **body mass index** | 978 | 13.15 |
| **Systolic blood pressure** | 957 | 12.87 |
| **Diastolic blood pressure** | 958 | 12.89 |
| **Fasting blood glucose** | 16 | 0.22 |
| **HbA1c** | 51 | 0.69 |
| **Total cholesterol** | 7 | 0.09 |
| **Triglycerides** | 7 | 0.09 |
| **low-density lipoprotein cholesterol** | 12 | 0.16 |
| **estimated glomerular filtration rate** | 18 | 0.24 |
| **Dyslipidaemia** | 113 | 1.52 |
| **Lipid-lowering drugs** | 117 | 1.57 |
