# Supplementary figures and images for: Predictive value of uric acid-to-high-density lipoprotein cholesterol ratio for cardiometabolic multimorbidity in middle-aged and older adults: A nationwide prospective cohort study
Source: Medicine (Baltimore). 2026 Jul 10;105(28):e49740. doi: 10.1097/MD.0000000000049740 (PMC13362854; doi:10.1097/MD.0000000000049740)

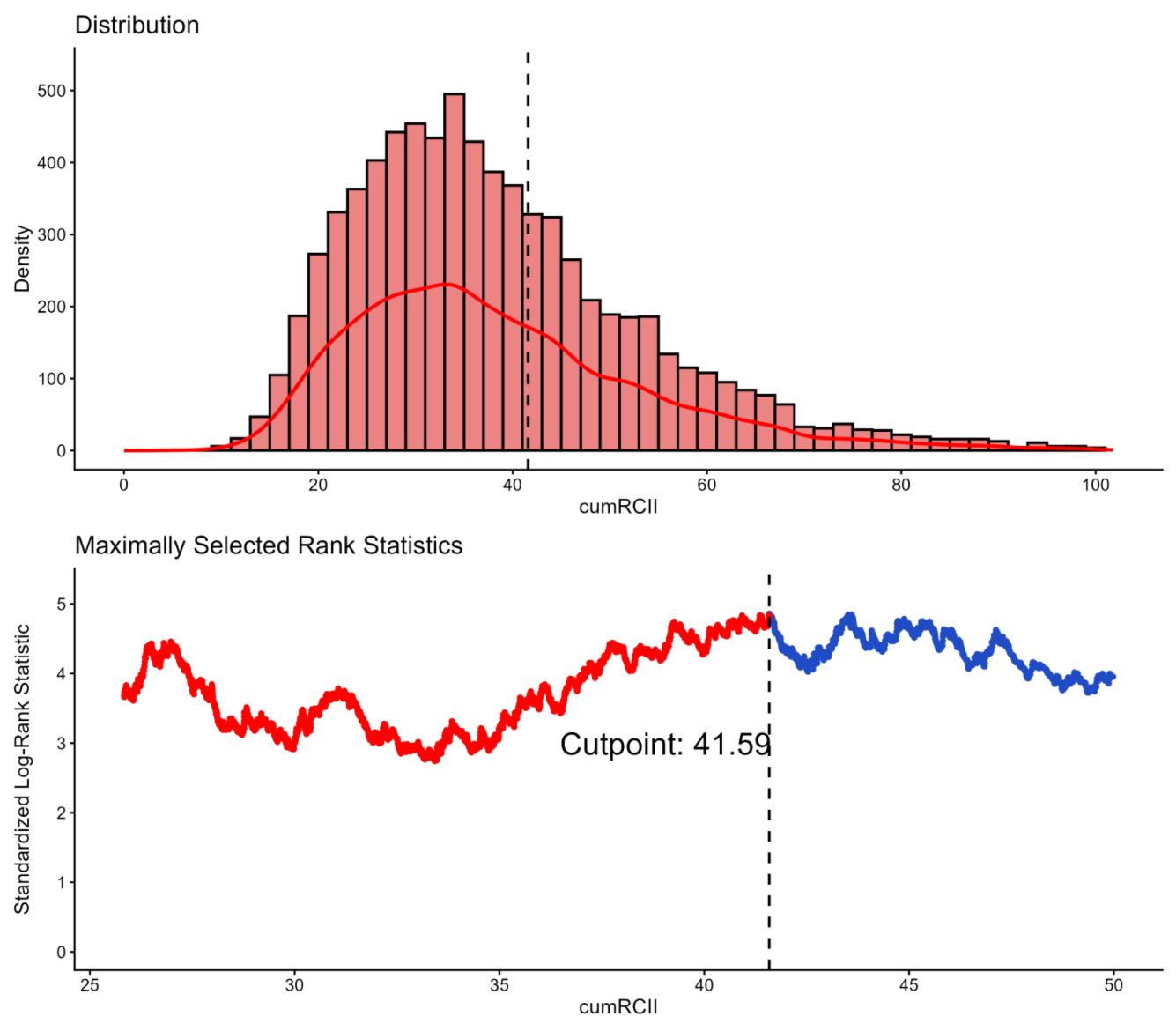

Supplement: Supplementary file 2 [file medi-105-e49740-s002.jpg]

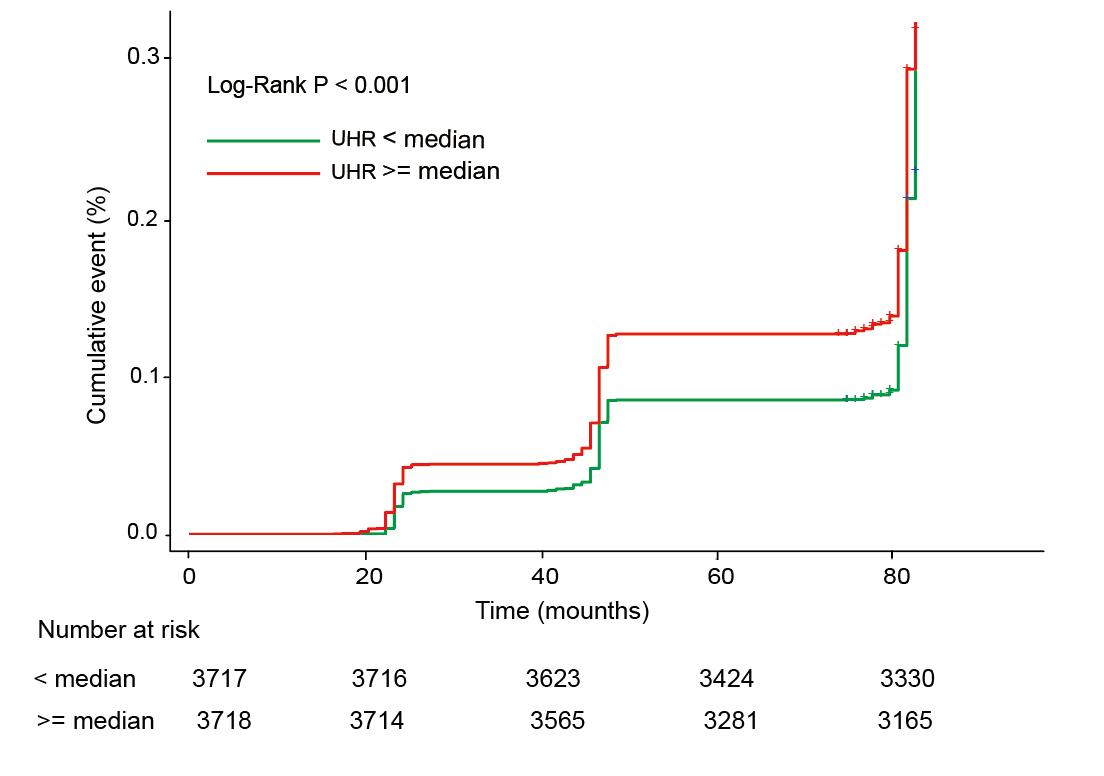

Supplement: Supplementary file 4 [file medi-105-e49740-s004.jpg]

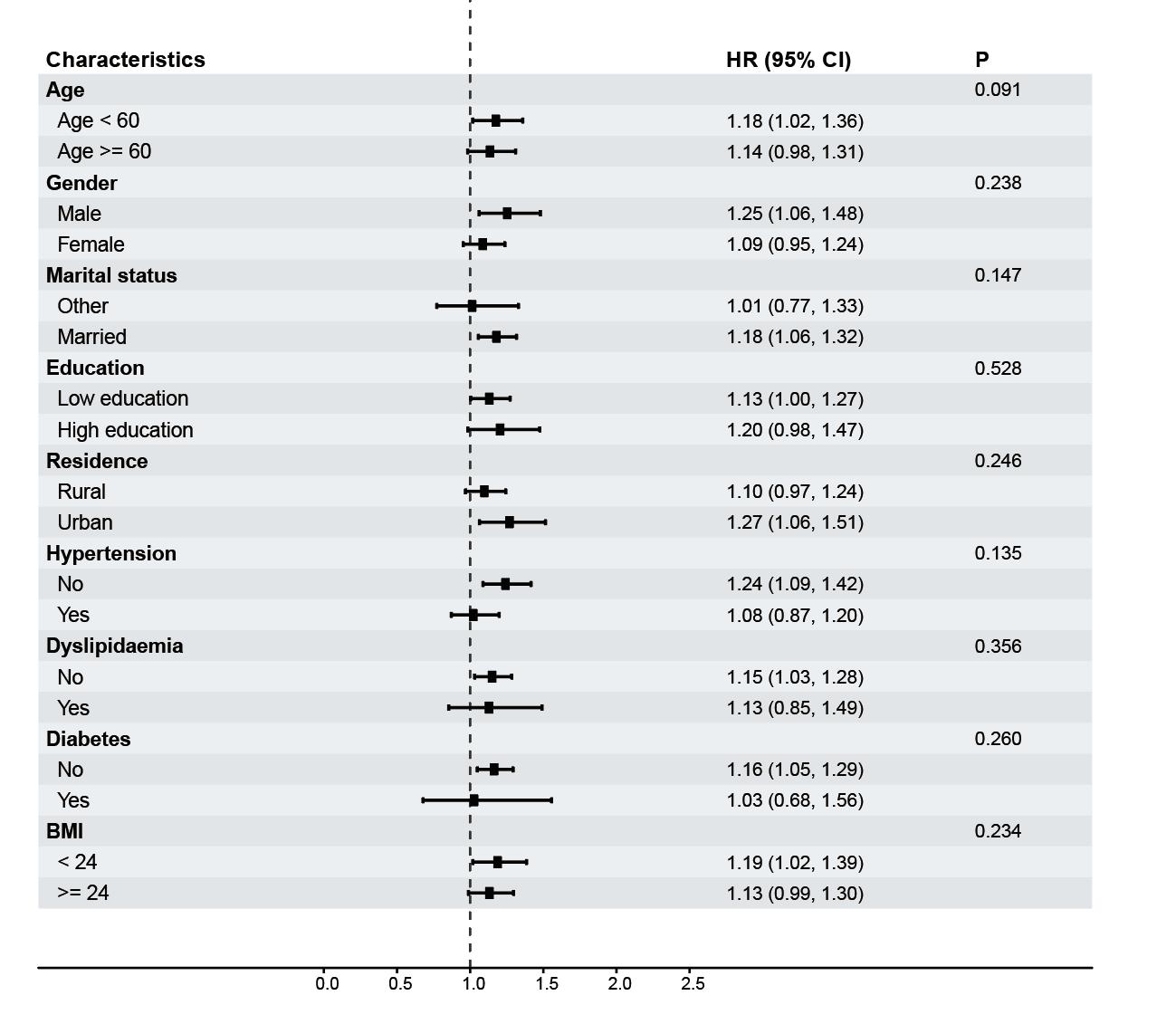

Supplement: Supplementary file 5 [file medi-105-e49740-s005.jpg]
