# Supplementary material for: Predictive value of uric acid-to-high-density lipoprotein cholesterol ratio for cardiometabolic multimorbidity in middle-aged and older adults: A nationwide prospective cohort study
Source: Medicine (Baltimore). 2026 Jul 10;105(28):e49740. doi: 10.1097/MD.0000000000049740 (PMC13362854; doi:10.1097/MD.0000000000049740)
Supplement: Supplementary file 3 [file medi-105-e49740-s003.docx]

**Table S2 Association between baseline UHR (median split) and CMM**

| **UHR** | **Model 1** | |  | **Model 2** | |  | **Model 3** | |
| --- | --- | --- | --- | --- | --- | --- | --- | --- |
|  | **HR (95% CI)** | ***P*** |  | **HR (95% CI)** | ***P*** |  | **HR (95% CI)** | ***P*** |
| **＜median** | Reference |  |  | Reference |  |  | Reference |  |
| **≥ median** | 1.50 (1.36–1.65) | <0.001 |  | 1.60 (1.45–1.76) | <0.001 |  | 1.16 (1.05–1.29) | 0.004 |
| ***P* value** |  | <0.001 |  |  | <0.001 |  |  | 0.004 |

**Notes:** Model 1 was unadjusted. Model 2 was adjusted for age, sex, education level, marital status, and residence. Model 3 was further adjusted for smoking status, alcohol consumption, body mass index, estimated glomerular filtration rate, and C-reactive protein.

**Abbreviations:** CI, confidence interval; HR, hazard ratio; UHR, uric acid-to-high-density lipoprotein cholesterol ratio.
