# Supplementary material for: Predictive value of uric acid-to-high-density lipoprotein cholesterol ratio for cardiometabolic multimorbidity in middle-aged and older adults: A nationwide prospective cohort study
Source: Medicine (Baltimore). 2026 Jul 10;105(28):e49740. doi: 10.1097/MD.0000000000049740 (PMC13362854; doi:10.1097/MD.0000000000049740)
Supplement: Supplementary file 6 [file medi-105-e49740-s006.docx]

**Table S3. Sensitivity analysis of the association between UHR and CMM**

| **UHR quartile** | **Model 1** | |  | **Model 2** | |  | **Model 3** | |
| --- | --- | --- | --- | --- | --- | --- | --- | --- |
|  | **HR(95%CI)** | ***P*** |  | **HR(95%CI)** | ***P*** |  | **HR(95%CI)** | ***P*** |
| **Q1** | Reference |  |  | Reference |  |  | Reference |  |
| **Q2** | 1.10 (0.95–1.27) | 0.196 |  | 1.13 (0.97–1.30) | 0.108 |  | 0.99 (0.85–1.14) | 0.841 |
| **Q3** | 1.40 (1.22–1.60) | <0.001 |  | 1.50 (1.30–1.73) | <0.001 |  | 1.11 (0.96–1.28) | 0.177 |
| **Q4** | 1.93 (1.68–2.22) | <0.001 |  | 1.76 (1.54–2.01) | <0.001 |  | 1.21 (1.05–1.40) | 0.011 |
| ***P* for trend** |  | <0.001 |  |  | <0.001 |  |  | 0.003 |

**Notes:** Model 1 was unadjusted. Model 2 was adjusted for age, sex, education level, marital status, and residence. Model 3 was further adjusted for smoking status, alcohol consumption, body mass index, estimated glomerular filtration rate, C-reactive protein, and lipid-lowering drug use

**Abbreviations:** CI, confidence interval; HR, hazard ratio; UHR, uric acid-to-high-density lipoprotein cholesterol ratio.
