# Supplementary material for: Predictive value of uric acid-to-high-density lipoprotein cholesterol ratio for cardiometabolic multimorbidity in middle-aged and older adults: A nationwide prospective cohort study
Source: Medicine (Baltimore). 2026 Jul 10;105(28):e49740. doi: 10.1097/MD.0000000000049740 (PMC13362854; doi:10.1097/MD.0000000000049740)
Supplement: Supplementary file 7 [file medi-105-e49740-s007.docx]

**Table S4. Sensitivity analysis of the association between CumUHR and CMM**

| **CumUHR** | **Model 1** | |  | **Model 2** | |  | **Model 3** | |
| --- | --- | --- | --- | --- | --- | --- | --- | --- |
|  | **HR(95%CI)** | ***P*** |  | **HR(95%CI)** | ***P*** |  | **HR(95%CI)** | ***P*** |
| Low | Reference |  |  | Reference |  |  | Reference |  |
| High | 1.52 (1.38-1.67) | <0.001 |  | 1.63 (1.47-1.79) | <0.001 |  | 1.18 (1.07-1.31) | 0.002 |
| *P* value |  | <0.001 |  |  | <0.001 |  |  | 0.002 |

**Notes:** Model 1 was unadjusted. Model 2 was adjusted for age, sex, education level, marital status, and residence. Model 3 was further adjusted for smoking status, alcohol consumption, body mass index, estimated glomerular filtration rate, C-reactive protein, and lipid-lowering drug use

**Abbreviations:** CI, confidence interval; HR, hazard ratio; UHR, uric acid-to-high-density lipoprotein cholesterol ratio.
